# Special Issue: Viruses Infecting Fish, Amphibians, and Reptiles

**DOI:** 10.3390/v3091609

**Published:** 2011-09-02

**Authors:** V. Gregory Chinchar

**Affiliations:** Department of Microbiology, University of Mississippi Medical Center, 2500 N. State Street, Jackson, MS 39216, USA; E-Mail: vchinchar@umc.edu; Tel.: +1-601-984-1743 Fax: +1-601-984-1708

Although viruses infecting and affecting humans are the focus of considerable research effort, viruses that target other animal species, including cold-blooded vertebrates, are receiving increased attention. In part this reflects the interests of comparative virologists, but increasingly it is based on the impact that many viruses have on ecologically and commercially important animals. Frogs and other amphibians are sentinels of environmental health and their disappearance following viral or fungal (chytrid) infection is a cause for alarm. Likewise, because aquaculture and mariculture are providing an increasingly large percentage of the “seafood” consumed by humans, viral agents that adversely impact the harvest of cultured fish and amphibians are of equal concern. In this special issue of Viruses, the authors provide a snapshot of selected viral agents responsible for disease among fish, amphibians, and reptiles. Moreover, because viral infection is intimately connected with host immunity (or its lack), two articles are devoted to immune responses in fish and amphibians. The remaining reports focus on either general aspects of viral infection in ectothermic vertebrates or a discussion of specific viruses, their replication, and their impact on their hosts. Through these reviews, we hope to make the reader aware of the impact of viruses on lower vertebrates and the ecological and commercial consequences of viral infection. From a practical (*i.e.,* applied) point of view, these studies may lead to development of vaccines that protect commercially important fish (and amphibians) from viral disease. In addition, these studies may shed light on the evolution of the immune response as lower vertebrates, separated from their warm-blooded brethren by as much as 400 million years have likely developed novel ways of combating viral pathogens.

